# Mobile phone problem use and depressive symptoms: the mediating role of social support and attitude to aging among Chinese older adults

**DOI:** 10.1186/s12888-024-05565-x

**Published:** 2024-02-16

**Authors:** Linlin Ding, Zhihan Li, Hao Jiang, Xiaona Zhang, Zhenfang Xiong, Xinhong Zhu

**Affiliations:** 1 School of Nursing, Hubei University of Chinese Medicine, #1 Huangjiahu West Road, Wuhan, 430065 China; 2 School of Nursing and Health Administration, Wuhan Donghu University, Wuhan, 430212 China; 3 Hubei Shizhen Laboratory, Wuhan, 430060 China

**Keywords:** Mobile phone problem use, Social support, Attitudes to aging, Depressive symptoms, Older adults

## Abstract

**Background:**

Little is known about mobile phone problem use (MPPU) among older adults. This study investigated critical factors affecting MPPU and filled the gap between MPPU and depressive symptoms in older people.

**Methods:**

A cross-sectional study was conducted in community (*n* = 376) with questionnaires of Multidimensional Scale of Perceived Social Support (MSPSS), Geriatric Depression Scale (GDS-15), Attitudes to Aging Questionnaire (AAQ) and Mobile Phone Problem Use Scale (MPPUS).

**Results:**

80.9% of older people used smartphones and spend less than three hours on mobile phone per day. The average MPPU score of Chinese elderly is greater than the cut off to 41. Female (β = -0.11, *P* = 0.037), living with spouse (β = -0.17, *P* = 0.03), and late marriage age (β = -0.16, *P* = 0.007) are less likely to develop MPPU. The relationship between MPPU and depressive symptoms was partially mediated by social support and attitude to aging.

**Conclusion:**

Elderly people generally have higher MPPU scores. MPPU was associated with depressive symptoms, through social support and attitude to aging.

## Introduction

During the past few years, there has been an obvious surge witnessed of the usage of mobile phones, from younger users to older users. It is demonstrated by the data of the National Bureau of Statistics, in 2018, the population of the elders with the age of 60 and above in China was 249 million, with the proportion of 17.9% of the total population [1]. Apart from that, according to the 50rd Statistical Report on Internet Development in China, there was 1,051 million smartphone internet users in 2021, with the percentage of elderly Internet users aged 60 and above was 11.3% [2]. The increase in mobile phone usage has two side effects. For instance, with the help of mobile phones, people can manage their work better, improve usability, and keep in touch with family and friends on a regular basis. In contrast, using a mobile phone can be problematic and have serious consequences [3]. Mobile Phone Problem Use (also known as mobile phone addiction, compulsive mobile phone use) have been documented in teens and young adults, with affected individuals experiencing unpleasant withdrawal symptoms when their phones are turned off or out of reach [4, 5]. It is suggested by most studies that children and adolescents are more likely to use their mobile phone excessively, which may pose negative impacts on their lives such as depression, isolation, deficiency of sleep, and lower academic performance [3, 6]. Specifically, MPPU may lead to financial problems, aggressive behavior (e.g., cyberbullying), self-reported addiction, and addiction-like symptoms (e.g., loss of cravings and control) [7]. A study from Norway shows that the proportion of mobile phone addiction among the elderly is low [8]. Norwegian older adults reside in a country that ranks highly on digitalization indexes [8]. Moreover, Norway is a developed country, and this situation may be different from that of China, a developing country. Therefore, due to rapid aging in China and the negative consequences of MPPU, it is necessary to investigate the MPPU among the elderly in China. It could be insightful to identify critical factors affecting MPPU and the negative consequences of MPPU among older people, especially in China.

The act of providing social support can be viewed as an allocation of social resources to each individual in an appropriate way since it is commonly believed that it can improve audience’s lives [9, 10]. There are a number of aspects to this concept, including structural support (e.g., size of the network, sources, and frequency of support) and functional supports (e.g., support for emotion, instruction, and information; satisfaction; and social connection). Researches show that MPPU affects smartphone users’ perceptions, causing them to overestimate the emotion gain of mobile phones and correspondingly underestimate its negative impacts [11, 12]. Therefore, we hypothesize as follows: MPPU will be negatively related to social support (H1). Furthermore, social support can promote the physical and mental health of the elderly. It can protect them from the harmful impacts on life’s events, such as widowhood and depression. It can also enhance their quality of life [10, 13, 14]. As a matter of fact, a previous study discover that social support with high quality and social networks of a large size actually decreases the chances of depressive symptoms in older Asians living in the community [15]. According to coping theory, a good social environment will strengthen the relationship between older adults’ positive attitudes toward ageing and their mental health [16]. Hence, we hypothesize as follows: Social support and attitude to ageing will be positively related (H2).

**Fig. 1:**
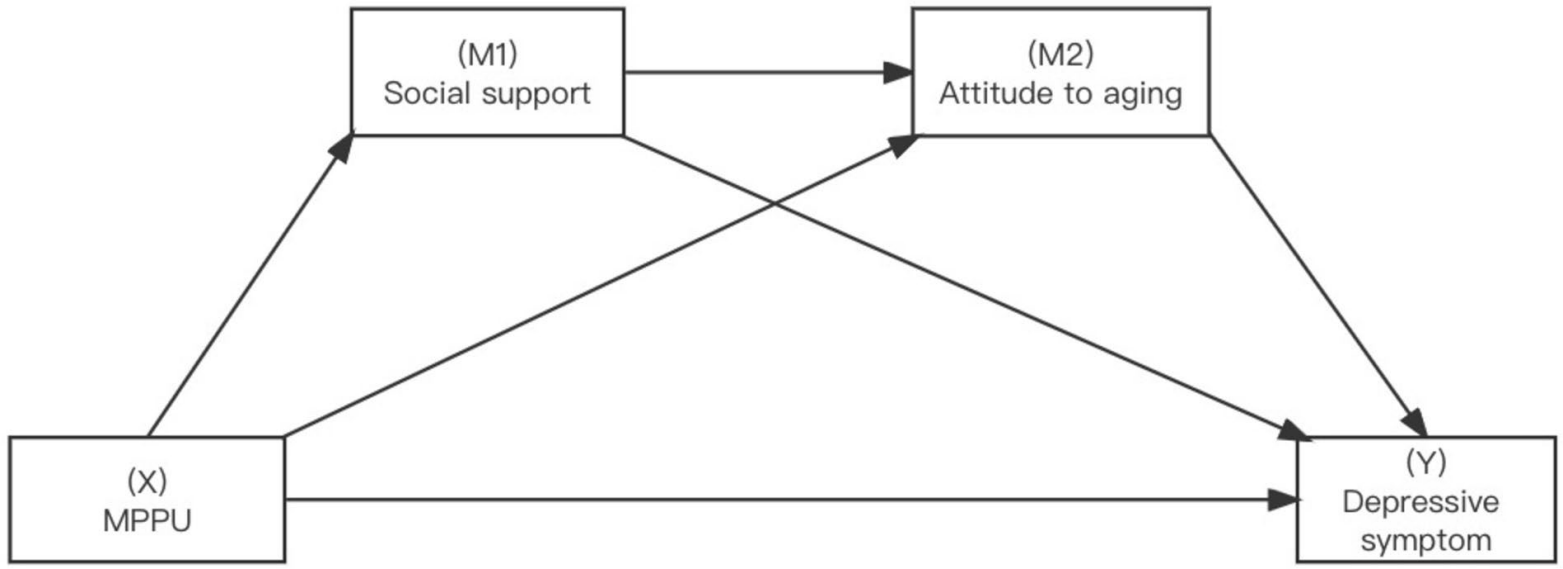
Hypothesized models of association involving MPPU, social support, attitude to aging, depressive symptoms

Attitudes toward ageing is a broad concept defined as experiences and perceptions of aging and the aging process. The positive aging attitude involves valuing wisdom, growth, and maturity. In contrast, negative attitudes toward aging involve perceptions of the physical, psychological, and social losses experienced during aging [17].Mental health problems faced by an increasing number of older adults are a global public health issue. Depression impairs physical, cognitive, and social functioning in older adults and is associated with increased risk of suicide and morbidity, indicating a high disease burden in this population [18]. Due to the one-child policy, China is facing the fastest aging population in history [19]. Therefore, it is necessary to examine the association between attitude to ageing and depression. Specifically, we hypothesized that positive attitude to ageing and depression will be negatively related (H3). The risk factors associated with MPPU, in both cognitive and socio-emotional way are comparable to those seen in other behavioral addictions like shopping and gambling [20]. These include pre-existing social anxiety and depression [21]. Research has shown that the nervous system is the most susceptible to the impact of mobile phone electromagnetic fields [22]. However, there is still insufficient research on the psychological mechanisms linking MPPU and depression in terms of mental health factors. As we mentioned earlier, MPPU increases depression in adolescents, however this link is unclear in older adults. The social situation and mentality of the elderly are very different from those of teenagers. Based on the relationship we discussed earlier among MPPU, social support, attitudes towards aging, and depressive symptom, it is imperative to better understand the mediating role of social support and attitude to aging between MPPU and depressive symptom in the elderly in order to improve their health. In accordance with these findings, we hypothesize as follows: MPPU will be associated with increased depressive symptom through decreased social support and positive attitude to aging (H4). We proposed a model in this study that is depicted graphically below (Fig. 1).

Although it has been a decade since mobile phones became popular, there has been little study on relationship between mobile phone problem use and depressive symptoms, espeacially the mediation role of social support and attitude to aging. This study was aimed to (1) Analyzing the actual status of mobile phone and social media usage among the elderly (2). Analyzing the predictive factors of MPPU (3). Analyzing the relational structure between MPPU and depressive symptoms mediated by social support and attitude to aging, as postulated in Fig. 1 in this study. Given to the large usage of the mobile phone and its potential adverse outcomes, it is of great significance for researchers to understand MPPU and figure out the association between MPPU and depressive symptom.

## Methods

### Sample and data collection

**Fig. 2:**
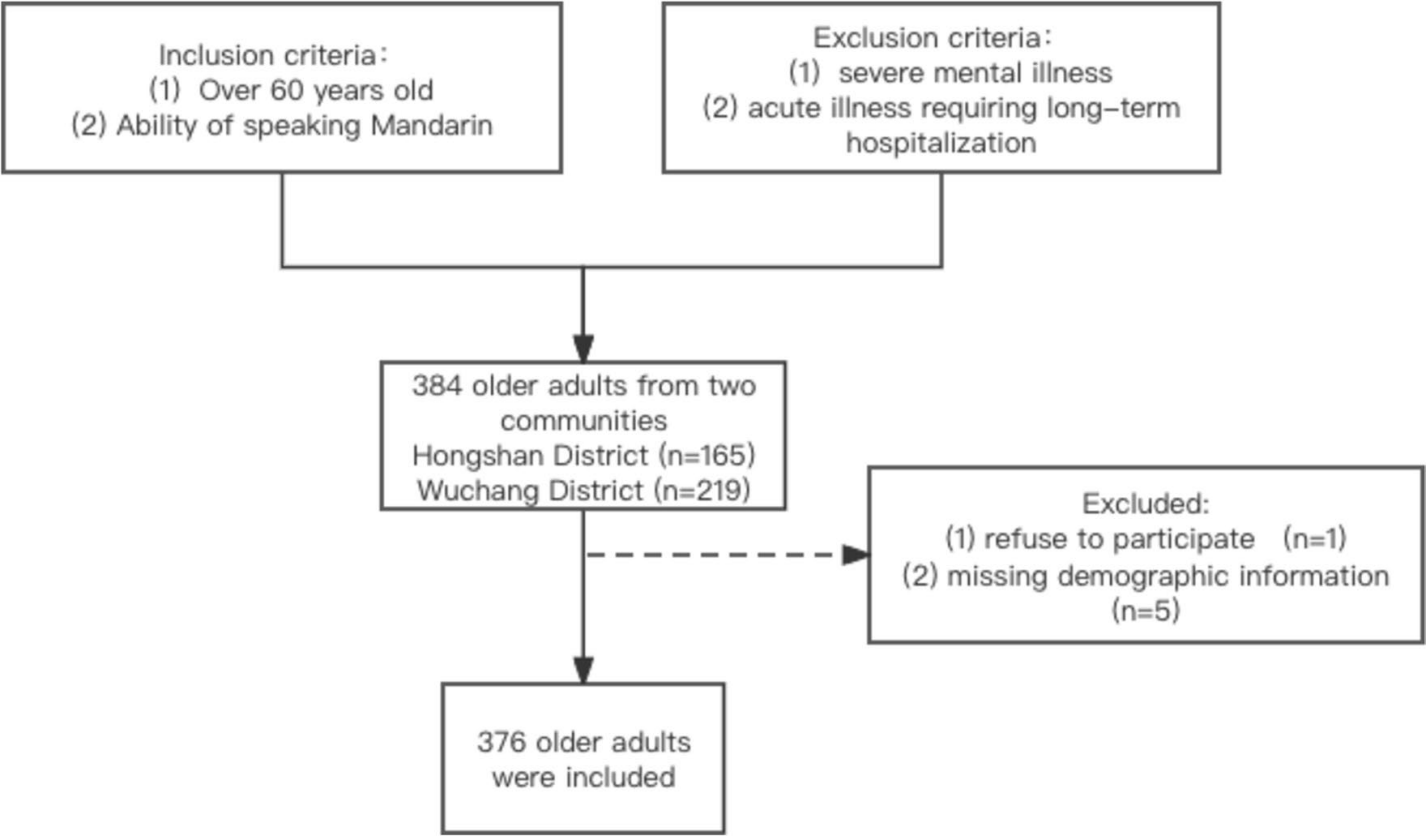
Flowchart for population selection

Three hundred and eighty-four older adults were recruited from two communities in Wuhan, Hubei, between January 2023 and February 2023. Wuhan is the capital of Hubei Province. Wuhan city includes thirteen districts. We selected two urban areas, namely the communities in Hongshan District and Wuchang District. These two districts are the main urban areas of Wuhan. Our questionnaires are collected and completed offline by researchers face-to-face. It was a voluntary study in which each participant participated. The criteria were the elders (1) aged over 60 and (2) mastery of Mandarin. It was not possible to include in the study older adults suffering from major psychiatric disorders or acute illnesses that required long-term hospitalization. There were 384 participants, but one refused to participate; therefore, there were 383 participants who signed the informed consent form. A total of 376 valid responses were analyzed after the missing information (*n* = 5) was excluded (Fig. 2).

### Instruments

#### An assessment of perceived social support on a multidimensional scale

A Chinese version of the Multidimensional Scale of Perceived Social Support (MSPSS) was used in this research to collect data on the perception of social support as well as how people feel about the support they receive from family, friends, and significant others on the basis of their perception of the value of their support [23, 24]. The questionnaire consists of 12 items, each of which is scored on a Likert scale of 1 to 5, from 1 (support is not available) to 5 (very supportive). An individual’s social support is measured on a scale of 12 to 60, with higher values indicating higher levels of social support. According to a previous study, the Cronbach’s alpha of this scale was 0.88, [23] and in this study, it reached 0.94.

#### Geriatric depression scale

Depressive symptoms were measured using a shortened Chinese version of the Geriatric Depression Scale (GDS-15) [25]. A total of 15 “Yes/No” questions are asked across emotional, cognitive, and behavioral dimensions. Values range from 0 to 15, where values of 0 - 4 indicate no symptoms of depression, 5 -10 indicate mild symptoms of depression and 11-15 indicate severe symptoms of depression [25]. According to the original scale, sensitivity was 80%, specificity was 75%, and Cronbach’s alpha was 0.80 [26]. In addition, a Chinese version of the GDS-15 with a cutoff point of 5 has been validated for culture variation and found to be valid and reliable for older adults in Taiwan [27, 28]. In this study, Cronbach’s alpha was 0.84.

#### Attitude to aging questionnaire

This questionnaire is designed as a means for individuals to express their views on the aging process, which is composed of 24 items categorized into 3 domains: Psychosocial loss (PL), physical change (PC), as well as psychological growth (PG) [29]. Cronbach’s alpha coefficients for all three sub-scales (0.68, 0.75, and 0.84), good test-retest reliability, and concurrent validity were established [29]. Psychiatric and social deficiencies associated with aging are the primary focus of the psychosocial loss domain. As we age, we observe and assess physical changes as well. Additionally, the psychological growth domain is intended to measure the positive aspects of wisdom and generativity that adults may experience as they age. In each domain there are eight items, which are based on self-reports with values ranging from 1 to 5, where a score of 1 indicates strong disagreement or not at all true, and a score of 5 indicates strong agreement or extremely accurate. To ensure consistency with the two other subscales, the psychosocial loss subscore was recoded in order to reflect a more positive attitude towards aging in each domain. A stronger endorsement of the predominant theme in each subscale was indicated by obtaining a higher score. This approach aligns with findings reported in various publications [29]. In order to solve the problem that there is no effective Chinese measurement method for attitude to aging, this study generates a Chinese version of AAQ through back translation. First, the research team translated AAQ into Chinese. The Chinese version is then translated into English by external professional translators. The team then compared the back-translated version with the original English version and obtained input from a panel of experts. In this study, the sub-scales displayed good reliability with Cronbach’s alphas exceeding 0.75 for all three sub-scales.

#### An assessment of the problematic use of mobile phones based on ten items

Chinese version of Foerster’s 10-item mobile phone problematic use scale (MPPUS-10) was used to measure MPPU [30]. In order to avoid questionnaire fatigue among respondents, we used short versions of measurement instruments. With a Cronbach’s alpha of 0.85, MPPUS-10 is highly representative of the original MPPUS-27 [31]. The MPPUS-10 includes “loss of control,” “withdrawal,” “negative influence in life,” “cravings” related to addictive behaviors, and “peer dependence” related to peer influence. Based on the results of the 10 items, a final sum score with a theoretical maximum range of 10-100 points will be calculated using a Likert scale ranging from 1 (not true at all) to 10 (extremely true) [31]. MPPUS-10 is a continuous scale that reflects the level of MPPU. Higher scores mean greater addiction to mobile phone use.

#### Mobile phone usage

Mobile phone usage was assessed with the following items: (1) Whether Use a Smartphone; (2) Why Don’t you Have a Smartphone; (3) Which Functions Do You Use often; (4) Purposes of using social media; (5) Which Kinds of Information do You Like; (6) Will you update; (7) Time spent on the phone; (8) What are the social media cohorts with which you interact most frequently; (9) Motivation for contacting these people; (10) Trouble Using Smart Phones; (11) Causes of Mobile Phone Disorders; (12) Check Your Phone Before Going to Bed; (13) Feeling at a loss if you forget your phone when you go out; (14) Do you often ask your loved ones about cell phone operation; (15) Difficulties You Have While Learning Smartphones.

#### Demographic questionnaire

Demographic information was also collected including basic information like gender, age, residential setting (rural or urban), living situation, education attainment (primary, junior, high, undergraduate, and above), marriage age, household income, payment type, and chronic diseases.

### Ethical consideration

This study followed the Declaration of Helsinki and it was reviewed and gained approval by the Human Ethics Committee of Hubei University of Chinese Medicine (NO.2022001). Participants were informed in advance of all information, including the study’s objectives and procedures and provided written consent. It was explained to them that they could withdraw from the research at any time.

### Data analysis/modeling

Descriptive statistics of the participants included demographics, mobile phone use, MSPSS, GDS-15, AAQ, and MPPUS-10. Linear regression models were fitted to identify significant factors (*p* < 0.05) related with MPPUS-10, which specified MPPU as the dependent, mobile phone usage, and demographics influencing factors as the independent variables.

Preliminary analyzes included bivariate assessments of associations between study variables and confirmatory factor analysis to test the MPPU, social support, attitudes toward aging and depressive symptoms.

In order to estimate the direct and indirect impacts on the path model (Fig. 1), Hayes (2012) used the PROCESS Macro (3.0) with bootstrapping standard errors, particularly, the direct effect of MPPU upon depressive symptoms as well as indirect impacts on MPPU on depressive via social support as well as attitudes toward aging. Using 5,000 bootstrapping simulations, Hayes’s PROCESS for SPSS Model 6 (Fig. 1) was employed to test the serial multiple mediation hypothesis [32]. Demographic information and mobile phone usage were included as control variables. Based on 5000 bootstrap samples, the standard errors of the impacts as well as their 95% bias-corrected Confidence Intervals (CI) were estimated. Overall model fit was assessed through multiple fit statistics, with the comparative fit index (CFI) ≥ 0.90, the root means square error of approximation (RMSEA) ≤ 0.06, and the standardised root mean square residual (SRMR) ≤ 0.08 indicating adequate fit [33]. All statistical analyses were conducted retrospectively using SPSS 26.0 and Amos 23.0. Statistical significance was determined in all cases by *P* < 0.05.

## Results

### Participants’ characteristics

In total, 376 adolescents owning a mobile phone were included in the baseline data analysis. 215 (57.2%) of the 376 participants were female and 161 (42.8%) males with a mean age of 69 years. A majority (57.4%) lived in urban areas, 51.1% of the participants lived with their spouses and 8.5% were still part of the active workforce (Table 1).

**Table 1:** General characteristics of the participants (*n* = 376)

Variables	n (%), mean ± SD	Variables	n (%), mean ± SD
**Sex**		**Household income**	
Male	161 (42.8)	< 3000 yuan	116 (30.9)
Female	215 (57.2)	3000 ~ 4999 yuan	177 (47.1)
**Age, mean ± SD**	69.2 ± 11.9	5000 ~ 7999 yuan	61 (16.2)
**Current residence**		≧ 8000yuan	22 (5.9)
Urban	216 (57.4)	**Payment type**	
Rural	160 (42.6)	Medical insurance	335 (89.1)
Living situation		At own expense	41 (10.9)
Living alone	65 (17.3)	**Chronic diseases**	
Living with spouse	192 (51.1)	Hypertension	226 (60.1)
Living with children and spouse	119 (31.6)	Diabetes	96 (25.5)
**Professional status**		Coronary heart disease	64 (17.0)
Employed	32 (8.5)	Stroke	27 (7.2)
Unemployed	344 (91.5)	Hepatopathy	28 (7.4)
**Level of education**		Others	130 (34.6)
Primary school	146 (38.8)	None	14 (3.7)
Junior school	128 (34.0)	**Relationship satisfaction**	
High school	62 (16.5)	Very good	158 (42.0)
Undergraduate students and above	40 (10.6)	Good	138 (36.7)
**Marriage age**		Moderately good	69 (18.4)
< 10 y	11 (2.9)	Not good	6 (1.6)
10 y ~ 19 y	7 (1.9)	Bad	5 (1.3)
20 y ~ 29 y	24 (6.4)		
≧30y	334 (88.8)		

### Mobile phone usage

Table 2 shows that of the mobile phone users, 80.9% were smartphone users. 13.8% thought the smartphone was difficult to learn and 29.8% of them spent 1 ~ 3 h per day using smartphone. Purposes for using smartphone were keeping in touch (71.0%), staying up to date with news and current events (51.6%), and killing the time (34.6%). Regarding the question “Contents preferences on smartphone”, 58.5% of participants reported hot social news, 48.9% of the participants reported friends’ updates and 41.5% reported health information. Regarding the question “The Most Interacted cohorts”, 90.2% reported children, 54.5% reported relatives, 50.8% reported friends, and 44.7% reported spouse. As for the question “Why Don’t you Have a Smartphone”, 52% reported it is difficult to learn, 28% reported it is rarely used and 24% reported nobody taught them.

**Table 2:** Self-assessed use of the mobile phone (*n* = 376)

Variables	n (%)	Variables	n (%)
**Whether use a smartphone**		**Who are the cohorts you interact most frequently on social media**	
Yes	304 (80.9)	Children	339 (90.2)
**Why don’t you have a smartphone**		Spouse	168 (44.7)
Dislike	22 (5.9)	Relatives	205 (54.5)
Economic problems	13 (3.5)	Friends	191 (50.8)
Rarely used	28 (7.4)	Colleagues	48 (12.8)
Difficult to learn	52 (13.8)	Medical worker	38 (10.1)
Security issues	21 (5.6)	Net friend	7 (1.9)
Nobody taught	24 (6.4)	**Motivation for contacting these people**	
None	2 (0.5)	Problems in life and family	342 (91.0)
**Which functions do you use often**		For job	69 (18.4)
Make a call	312 (83.0)	Consulting	85 (22.6)
Text	162 (43.1)	Maintain relationship	191 (50.8)
Photograph	136 (36.2)	**Trouble using smart phones**	
Weather forecast	164 (43.6)	Use of health code	128 (34.0)
Social media	199 (52.9)	No electronic registration	124 (33.0)
Game	41 (10.9)	Cannot use taxi app	124 (33.0)
E-books	47 (12.5)	No face recognition	120 (31.9)
**Purposes of using social media**		Difficulty in mobile payment	147 (39.1)
To stay up to date with news and current events	194 (51.6)	Difficulty shopping online	129 (34.3)
Learning	61 (16.2)	Cannot use social software	71 (18.9)
Entertainment	76 (20.2)	Education of left-behind children	41 (10.9)
Keep in touch	267 (71.0)	No problem	70 (18.6)
Shopping	67 (17.8)	**Causes of mobile phone disorders**	
Game	48 (12.8)	Inadequate social facilities	111 (29.5)
Kill the time	130 (34.6)	Lack of technical products for the elderly	221 (58.8)
Because my friends use it too	44 (11.7)	The society does not care enough about the elderly	142 (37.8)
Share photos or videos	73 (19.4)	Neglect of online media	74 (19.7)
Start a topic	17 (4.5)	**Check your phone before going to bed**	
Chase the trend	30 (8.0)	Extremely true	75 (19.9)
Out of curiosity	41 (10.9)	Very true	73 (19.4)
**Which kinds of information do you like**		Moderately true	111 (29.5)
Friends’ updates	184 (48.9)	Slightly true	83 (22.1)
Hot social news	220 (58.5)	Not at all true	34 (9.0)
Movies or TV shows	63 (16.8)	**Feeling at a loss if you forget your phone when you go out**	
Life service consulting	155 (41.2)	Extremely true	99 (26.3)
Knowledge that is useful to me	104 (27.7)	Very true	74 (19.7)
Health information	156 (41.5)	Moderately true	104 (27.7)
Sports event	30 (8.0)	Slightly true	64 (17.0)
Product information	28 (7.4)	Not at all true	35 (9.3)
Money stock	18 (4.8)	**Do you often ask your loved ones about cell phone operation**	
**Will you update**		Extremely true	94 (25.0)
No	240 (63.8)	Very true	142 (37.8)
Yes	136 (36.2)	Moderately true	86 (22.9)
**Time(h/day)**		Slightly true	38 (10.1)
< 1	99 (26.3)	Not at all true	16 (4.3)
1 ~ 3	112 (29.8)	**Difficulties you have while learning smartphones**	
3 ~ 5	98 (26.1)	Memory loss, repeated learning	154 (41.0)
≧5	67 (17.8)	Poor understanding	157 (41.8)
		Rejection of electronic products	61 (16.2)
		Worry about safety	151 (40.2)
		Vision problems	148 (39.4)
		Illiterate	41 (10.9)
		Inflexible fingers	69 (18.4)
		No problem	36 (9.6)

### Preliminary analyses

Intercorrelations between observed study variables (Pearson’s r) are reported in Table 3. All variables were significantly correlated, with effect sizes ranging from -0.495 (depressive symptoms and physical change) to 0.539 (psychological growth and psychosocial loss).

**Table 3:** Intercorrection matrix of obeserved study variables

	M	SD	1`	2	3	4	5	6
1. MPPU	41.7	15.09						
2. Social support	56.37	10.18	-.385^*^					
3. AAQ - Psychosocial loss	21.64	5.81	-.353^*^	.407^*^				
4. AAQ - Psychological growth	25.02	5.23	-.366^*^	.335^*^	.539^*^			
5. AAQ - Physical change	26.13	5.62	-.329^*^	.452^*^	.565^*^	.508^*^		
6. Depressive symptoms	3.17	2.84	.387^*^	-.468^*^	-.448^*^	-.395^*^	-.495^*^	

^*^P < 0.01

### Factors influencing mobile phone usage

As depicted in Table 4, female (β = -0.11, *P* = 0.037), living with spouse (β = -0.17, *P* = 0.03), and late marriage age (β = -0.16, *P* = 0.007) are less likely to develop MPPU. Older people (β = 0.13, *P* = 0.018), and unemployed people (β = 0.15, *P* = 0.03) are more likely to develop MPPU. The reason of “security issues” (β = 0.11, *P* = 0.023) and “nobody taught” (β = 0.13, *P* = 0.018) for not having a smartphone, purposes of using smartphone for sharing photos or videos (β = 0.15, *P* = 0.023), updating (β = 0.17, *P* = 0.014), the amount of time spent on a mobile device (β = 0.21, *P* = 0.008), causes of “inadequate social facilities” (β = 0.11, *P* = 0.04) and “the society does not care enough about the elderly” (β = 0.1, *P* = 0.044) for mobile phone disorders, feeling at a loss if you forget your phone when you go out (β = -0.2, *P* = 0.001) in Model 2.

**Table 4:** Factors associated with MPPUS-10 among the elderly

Variables, n (%)	Model 1		Model 2	
	β (95%CI)	*P*	β (95%CI)	*P*
Sex				
Female (male)	-0.02 (-3.51-2.44)	0.725	-0.11 (-6.27-0.20)*	0.037
Age, mean ± SD	0.09 (-0.02-0.25)	0.094	0.13 (0.03-0.30)*	0.018
Current residence				
Rural (City)	0.13 (0.61-7.04)*	0.02	0.01 (-3.01-3.48)	0.886
Living situation				
Living with spouse (Living alone)	-0.12 (-7.88-0.76)	0.106	-0.17 (-9.66-0.51)*	0.03
Living with children and spouse (Living alone)	-0.14 (-8.84-0.07)	0.054	-0.15 (-9.52-0.49)*	0.03
Professional status				
Unemployed (Employed)	0.09 (-0.50-9.83)	0.077	0.15 (1.94-12.87)**	0.008
Level of education				
High school and above (Primary and Junior school)	0.11 (-0.04-3.46)	0.055	0.04 (-1.26-2.33)	0.558
Marriage Age	-0.28 (-9.67-4.41)***	0	-0.16 (-6.76-1.10)***	0.007
Household Income	0.05 (-1.04-2.88)	0.354	-0.04 (-2.68-1.34)	0.514
Payment type				
At own expense (medical insurance)	0.07 (-1.12-8.32)	0.135	-0.04 (-6.91-2.79)	0.404
Chronic diseases				
Hypertension	-0.05 (-4.93-1.75)	0.35	-0.06 (-5.09-1.45)	0.275
Diabetes	-0.06 (-5.51-1.27)	0.219	-0.02 (-3.86-2.82)	0.759
Coronary heart disease	-0.10 (-7.92-0.11)	0.057	-0.10 (-7.75-0.08)	0.055
Stroke	0.00 (-5.68-6.19)	0.933	0.04 (-3.23-8.16)	0.394
Hepatopathy	0.07 (-1.78-9.84)	0.173	0.03 (-4.13–7.22)	0.593
Others	-0.17 (-8.86-1.98) **	0.002	-0.13 (-7.86-0.28)*	0.035
None	-0.06 (-13.08-3.38)	0.248	-0.08 (-14.42-2.36)	0.158
Relationship Satisfaction	0.00 (-1.71-1.66)	0.979	0.01 (-1.64-1.86)	0.902
**Why don’t you have a smartphone**				
Security issues			0.11 (1.02-13.80) *	0.023
Nobody taught			0.13 (1.36-14.33) *	0.018
**Purposes of using social media**				
Share photos or vedios			0.15 (0.79-10.51) *	0.023
**Will you update**				
Yes			0.17 (0.72-6.41) *	0.014
Time(h/day)			0.21 (0.75-4.89) **	0.008
**Causes of mobile phone disorders**				
Inadequate social facilities			0.11 (0.16-7.02) *	0.04
The society does not care enough about the elderly			0.10 (0.08-6.23) *	0.044
Feeling at a loss if you forget your phone when you go out			-0.20 (-3.81-0.94) **	0.001
*R*^2^	0.193		0.519	
Adjusted *R*^2^	0.152		0.364	

Model 2: Adjusted mobile phone use

^*^P < 0.05

^**^P < 0.01

^***^P < 0.001

### Examination of hypotheses

The hypothesised model resulted in acceptable for the data in AAQ - psychosocial loss (CFI = 0.992; RMSEA = 0.018; SRMR = 0.033), AAQ – physical change (CFI = 0.989; RMSEA = 0.021; SRMR = 0.035) and AAQ – psychological growth (CFI = 0.989; RMSEA = 0.021; SRMR = 0.032). In sum, it explained 40% of the variance in AAQ - psychosocial loss, 42% of the variance in AAQ – physical change and 39% of the variance in AAQ – psychological growth.

**Fig. 3:**
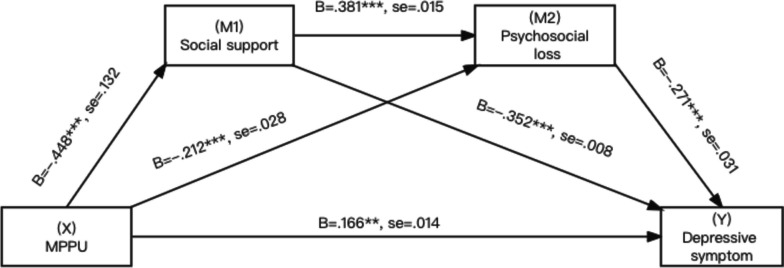
Association involving MPPU, social support, AAQ - psychosocial loss, depressive symptoms

**Fig. 4:**
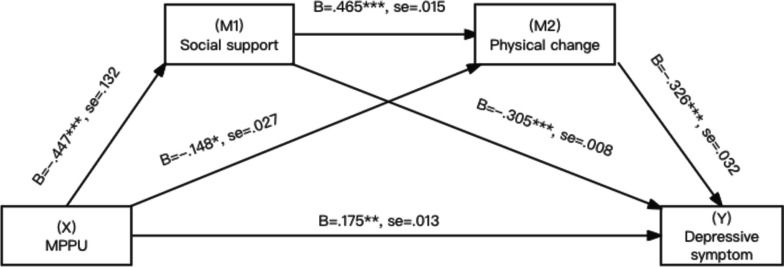
Association involving MPPU, social support, AAQ - physical change, depressive symptoms

**Fig. 5:**
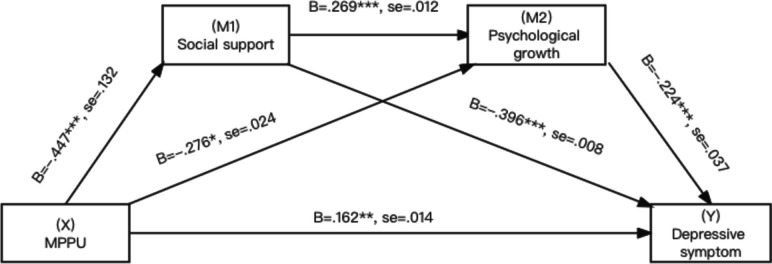
Association involving MPPU, social support, AAQ - psychological growth, depressive symptoms

As shown in Figs. 3, 4 and 5, MPPU was negatively associated with social support (β = -0.956, B = -0.448,SE = 0.132, t = -7.257, *p* < 0.001, lower limit confidence interval [LLCI] = -0.541; upper limit confidence interval [ULCI] = -0.348), supporting Hypothesis 1.

Next, social support was positively associated with all three factors of attitudes to ageing (β = 0.084, B = 0.381, SE = 0.015, t = 5.677, *p* < 0.001, lower limit confidence interval [LLCI] = 0.264; upper limit confidence interval [ULCI] = 0.493) in AAQ—psychosocial loss, (β = 0.101, B = 0.465, SE = 0.015, t = 6.735, *p* < 0.001, lower limit confidence interval [LLCI] = 0.351; upper limit confidence interval [ULCI] = 0.563) AAQ – physical change, (β = 0.048, B = 0.269, SE = 0.012, t = 4.006, *p* < 0.001, lower limit confidence interval [LLCI] = 0.146; upper limit confidence interval [ULCI] = 0.394) in AAQ- psychological growth, supporting Hypothesis 2.

All three factors were negatively associated with depressive symptoms (β = -0.140, B = -0.271, SE = 0.031, t = -4.533, *p* < 0.001, lower limit confidence interval [LLCI] = -0.378; upper limit confidence interval [ULCI] = -0.153) in AAQ—psychosocial loss, (β = -0.168, B = -0.326, SE = 0.032, t = -5.207, *p* < 0.001, lower limit confidence interval [LLCI] = -0.442; upper limit confidence interval [ULCI] = -0.207) in AAQ – physical change, (β = -0.142, B = -0.224, SE = 0.037, t = -3.830, *p* < 0.001, lower limit confidence interval [LLCI] = -0.338; upper limit confidence interval [ULCI] = -0.110) in AAQ- psychological growth, supporting Hypothesis 3.

The estimation of the indirect effect through both mediators was particularly relevant to Hypothesis 4. Evidence of a significant serial multiple mediator association can be obtained when a bias-corrected bootstrap confidence interval for the product of this path, which excludes zero, is considered [34]. The path from MPPU to depressive symptoms was indeed significant through both mediators with a significant point estimate for the effect of 0.046 and a 95% CI between 0.025 and 0.08 in psychosocial loss, the effect of 0.068 and a 95% CI between 0.041 and 0.108 in physical change and the effect of 0.027 and a 95% CI between 0.012 and 0.053 in psychological growth. Hypothesis 4 was supported. The direct, indirect, and total associations are presented in Table 5.

**Table 5:** Total, direct and indirect associations

Total, direct and indirect associations	Depressive symptoms as criterion			
	Effect	SE	Lower bound	Upper bound
**AAQ – Psychosocial loss**				
Total association of X on Y	0.427	0.049	0.325	0.515
Direct association of X on Y	0.166	0.057	0.053	0.273
X → M1 → Y	0.158	0.033	0.098	0.23
X → M2 → Y	0.057	0.02	0.025	0.108
X → M1 → M2 → Y	0.046	0.013	0.025	0.08
Total indirect effect	0.261	0.035	0.195	0.335
**AAQ – Physical change**				
Total association of X on Y	0.427	0.049	0.325	0.515
Direct association of X on Y	0.175	0.055	0.065	0.281
X → M1 → Y	0.136	0.034	0.074	0.206
X → M2 → Y	0.048	0.02	0.015	0.099
X → M1 → M2 → Y	0.068	0.017	0.041	0.108
Total indirect effect	0.252	0.035	0.187	0.323
**AAQ - Psychological growth**				
Total association of X on Y	0.427	0.049	0.325	0.515
Direct association of X on Y	0.162	0.059	0.046	0.275
X → M1 → Y	0.177	0.034	0.116	0.249
X → M2 → Y	0.062	0.021	0.029	0.114
X → M1 → M2 → Y	0.027	0.01	0.012	0.053
Total indirect effect	0.266	0.037	0.197	0.345

X = MPPU, M1 = social support, M2 = attitude to aging, Y = depressive symptoms. Covariates included demographic characteristics. Number of bootstrap samples for bias corrected bootstrap confidence intervals: 5,000. Level of confidence for all confidence intervals: 95%

## Discussions

The current research aimed to explore mobile phone usage among older adults, including their social support, attitudes to aging, depressive symptoms, and MPPU, and the association involving these constructs. The average MPPU score of Chinese elderly is greater than the cut off to 41 [8]. Besides, regarding the relationships of these, the MPPU was sequentially and negatively associated with decreased social support. Social support was positively with attitude to aging, which was negatively related to increased depressive symptoms. That is, the MPPU was positively associated with depressive symptoms through decreased social support and attitude to aging.

Most of the participants used smartphones and the reasons for not using smartphones were difficult to learn, rarely used and nobody taught, but large proportions of them did not update their social media accounts. Researchers have examined how various types of smartphone applications, such as social media and games, can contribute to problematic smartphone usage in a variety of settings [3, 35]. Moreover, most participants reported that the purposes for using social media were for staying up-to-date with news and killing the time. An increase in the perception of emotional gain can result from the use of smartphones in compensating for emotional and psychological issues including loneliness [36]. The most interesting content on social media was hot social news and friends’ updates, answered by most of the participants. In the research, the amount of time spent on social media by most older users on a daily basis is in line with other survey results [8].

In addition, female, living with spouse, and late marriage age are associated with MPPU. Female are is positively associated with an increase in MPPU, which were reported in previous studies [30, 37, 38]. Previous research has shown that women use their phones more for social activities, while men use their phones more for gaming or business activities [39]. However, the confounding factor of different emotions brought about by gender cannot be ruled out. Older people and those without jobs may experience more loneliness. Previous research has shown that loneliness is positively linked to MPPU through emotion gain [8].

We postulated a model analyzing the mediated association involving MPPU and depressive symptoms, and it was mediated by social support and attitude to aging. Our study showed that MPPU was negatively linked to social support. Excessive use of mobile phones leads to a decrease in their ability to perceive emotions and thus their ability to perceive social support [8]. Moreover, as expected, social support was positively associated with attitude to aging, and attitude to aging was negatively linked to depression. The support from family, friends, and governmental programs not only effectively protected individuals from depressive symptoms but also mitigated the negative impacts of internalized negative stereotypes about aging. Positive attitudes toward aging helped to boost more confidence in dealing with the challenges as well as changes in their daily lives [40], thus reducing their psychological resistance to adopting mobile phones to compensate for deficits. Moreover, a large cross-sectional study on Chinese elders revealed that those with a more positive attitude towards aging reported better mental health outcomes [41]. Based on cognitive theory [42], it is suggested that older adults who internalize negative attitudes towards aging tend to engage in selective rumination, directing their attention towards the negative aspects of the aging process. Consequently, individuals with negative aging attitudes are more prone to experiencing depression [43].

It is also worth noting that the serial multiple mediational model yielded a direct and positive association between MPPU and depressive symptoms. This study confirmed MPPU was negatively relevant to depression symptoms, consistent with the findings of previous overseas studies [38, 44–46]. This may be due to mobile phone addiction, which immerses them in the online world and leads to less offline activities and communication, which has a negative impact on their mood. The effect of this was found to account for 39%- 42% of the total effect. As a result, an obvious conclusion has been drawn that intervention on depression of the elders should be focused on factor of social support and mobile phone usage.

## Limitations

There were some limitations to this study. First, our sample only included smartphone users living in two communities in Wuhan, which may affect the generalizability of our results. Second, because the participants were informed that the study would be examining social support, depressive symptoms, attitude to aging, and MPPU, reporting bias may be likely to occur. Third, the study focused on social support and attitude to aging that may mediate the association involving MPPU and depressive symptoms, while omitting other possible mediators, such as self-control, and Fear of Missing Out (FoMO). Future studies should cover these above variables. Fourth, a cross-sectional design did not provide support for the causality among studied variables postulated in this study. Finally, with the deficiency of relevant data, factors like characteristics of individual that is connected to negative aging perceptions and trend of frustration were not taken into consideration.

## Conclusion

This study highlights that elderly people have MPPU scores above the cut off value.. Moreover, MPPU was associated with gender, age, living situation, professional status and marriage age. Above all, the impacts on MPPU upon depressive symptoms receive mediation from social support and attitude to aging.

## Data Availability

The datasets used and/or analysed during the current study available from the corresponding author on reasonable request.

## Abbreviations

MPPU: Mobile Phone Problem Use
MSPSS: Multidimensional Scale of Perceived Social Support
GDS-15: Geriatric Depression Scale
AAQ: Attitudes to Aging Questionnaire
FoMO: Fear of Missing Out
